# Variability in prostate cancer detection among radiologists and urologists using MRI fusion biopsy

**DOI:** 10.1002/bco2.294

**Published:** 2023-10-08

**Authors:** Hiten D. Patel, Whitney R. Halgrimson, Sarah E. Sweigert, Steven M. Shea, Thomas M. T. Turk, Marcus L. Quek, Alex Gorbonos, Robert C. Flanigan, Ari Goldberg, Gopal N. Gupta

**Affiliations:** ^1^ Department of Urology Loyola University Medical Center Maywood Illinois USA; ^2^ Department of Urology, Feinberg School of Medicine Northwestern University Chicago Illinois USA; ^3^ Department of Radiology Loyola University Medical Center Maywood Illinois USA

**Keywords:** magnetic resonance imaging, practice variation, prostate biopsy, prostate cancer, prostate cancer detection

## Abstract

**Objectives:**

The aim of this study is to evaluate the impact of radiologist and urologist variability on detection of prostate cancer (PCa) and clinically significant prostate cancer (csPCa) with magnetic resonance imaging (MRI)‐transrectal ultrasound (TRUS) fusion prostate biopsies.

**Patients and methods:**

The Prospective Loyola University MRI (PLUM) Prostate Biopsy Cohort (January 2015 to December 2020) was used to identify men receiving their first MRI and MRI/TRUS fusion biopsy for suspected PCa. Clinical, MRI and biopsy data were stratified by radiologist and urologist to evaluate variation in Prostate Imaging‐Reporting and Data System (PI‐RADS) grading, lesion number and cancer detection. Multivariable logistic regression (MVR) models and area under the curve (AUC) comparisons assessed the relative impact of individual radiologists and urologists.

**Results:**

A total of 865 patients (469 biopsy‐naïve) were included across 5 urologists and 10 radiologists. Radiologists varied with grading 15.4% to 44.8% of patients with MRI lesions as PI‐RADS 3. PCa detection varied significantly by radiologist, from 34.5% to 66.7% (*p* = 0.003) for PCa and 17.2% to 50% (*p* = 0.001) for csPCa. Urologists' PCa diagnosis rates varied between 29.2% and 55.8% (*p* = 0.013) and between 24.6% and 39.8% (*p* = 0.36) for csPCa. After adjustment for case‐mix on MVR, a fourfold to fivefold difference in PCa detection was observed between the highest‐performing and lowest‐performing radiologist (OR 0.22, 95%CI 0.10–0.47, *p* < 0.001). MVR demonstrated improved AUC for any PCa and csPCa detection when controlling for radiologist variation (*p* = 0.017 and *p* = 0.038), but controlling for urologist was not significant (*p* = 0.22 and *p* = 0.086). Any PCa detection (OR 1.64, 95%CI 1.06–2.55, *p* = 0.03) and csPCa detection (OR 1.57, 95%CI 1.00–2.48, *p* = 0.05) improved over time (2018–2020 vs. 2015–2017).

**Conclusions:**

Variability among radiologists in PI‐RADS grading is a key area for quality improvement significantly impacting the detection of PCa and csPCa. Variability for performance of MRI‐TRUS fusion prostate biopsies exists by urologist but with less impact on overall detection of csPCa.

## 1 INTRODUCTION

An estimated 248 530 new cases of prostate cancer were diagnosed in 2021, with an increasing role played by magnetic resonance imaging (MRI) to aid in identification and localisation of clinically significant prostate cancer (csPCa).^1^ The PROMIS trial proposed the use of MRI as a triage test which could theoretically avoid up to 27% of primary biopsies with 93% sensitivity of csPCa.^2^ The PRECISION and PRECISE trials demonstrated improved detection of csPCa between 5% and 12% with MRI‐targeted biopsy over transrectal ultrasound (TRUS) template biopsy and improved exclusion of Gleason Grade 1 (GG1) disease by 12%.^3^, ^4^ A combined technique with MRI‐targeted and standard template biopsy in a real‐world cohort improved detection of csPCa by 10% although reduction in GG1 diagnoses was minimal at 0.5% over standard template biopsy, while the Trio study showed the addition of targeted biopsy upgraded 12.7% of cases to GG ≥ 2.^5^, ^6^ Use of MRI with combined targeted and template biopsy in biopsy‐naïve patients is now a guideline recommendation.^7^ However, given the use of MRI as a triage test or to guide prostate biopsy, variability in MRI performance at predicting csPCa is a common criticism and suggests opportunity for improvement.^8^, ^9^, ^10^

The Prostate Imaging‐Reporting and Data System (PI‐RADS) grading system provides a standardised interpretation paradigm to predict the presence of csPCa for a specific lesion in the prostate gland, with the intent to improve both diagnostic performance and reproducibility between radiologists.^11^ Despite widespread adoption of PI‐RADS grading, there appears to be a broad range of radiologists' interpretations of prostatic lesions. Furthermore, the positive predictive value of MRI for csPCa varies significantly across institutions, between 27% and 48% (interquartile range) for PI‐RADS scores ≥3.^12^

Therefore, the primary goal of this study was to assess variability in PI‐RADS classification across radiologists and the relative impact of variability between radiologists and urologists on prostate cancer detection.

## 2 METHODS

### 2.1 Study design

The study included men who underwent their first MRI for clinical suspicion of prostate cancer followed by transrectal MRI‐TRUS fusion prostate biopsy between January 2015 and December 2020. Patient records were abstracted retrospectively from the Prospective Loyola University Multiparametric MRI (PLUM) prostate biopsy cohort. All cases were performed at a single tertiary academic referral centre. Men who failed to have a biopsy or who had prior prostate cancer diagnosis were excluded. Cases were grouped by individual radiologist interpreting MRI and urologist performing prostate biopsy; individuals with <10 cases were included in the sample but excluded from performance comparisons. The Institutional Review Board approved the research protocol with informed consent waived for participants.

The primary study outcome was prostate cancer detection stratified by (1) radiologists performing PI‐RADS grading on prostate MRI and (2) urologists performing the prostate biopsy. Secondary outcomes examined the relative distribution of PI‐RADS lesions and number of lesions on MRI.

### 2.2 Study procedures

MRI was performed using 3‐Tesla MRI (Siemens Magnetom Triop and Verio). In rare instances, 1.5‐Tesla MRI (GE Optima MR450W) was employed when use of 3‐T coil was contraindicated. An endorectal coil was used for cases prior to 2019, after which it was routinely omitted for most patients. Postimage processing used DynaCAD software (Philips Healthcare, Best, Netherlands). MRI images were graded by experienced but nondedicated faculty using PI‐RADS version 2.0 or 2.1.^11^

All MRI‐TRUS fusion biopsies were performed transrectally by experienced urologists using the UroNav system (Invivo, Philips Healthcare). Transperineal biopsy was introduced after the study period. Standard template biopsies included two biopsy cores taken from each sextet region. Targeted biopsies were obtained at the discretion of the urologist; PI‐RADS 3‐5 lesions were routinely biopsied with two cores, while PI‐RADS 2 lesions were rarely sampled.

### 2.3 Study variables

Standard clinical variables of interest included age, race, family history of prostate cancer, abnormal digital rectal exam for prostate cancer, history of prior negative prostate biopsy and prostate‐specific antigen (PSA). PSA density was calculated using volume derived from MRI. Imaging parameters included estimated prostate volume, multifocality (number of lesions with PI‐RADS ≥3) and the highest PI‐RADS grade per study. Pathology data included Gleason Grade Group and the biopsy method.

### 2.4 Statistical analyses

Highest PI‐RADS lesion categorisation and number of lesions on MRI were stratified by radiologist and by urologist to evaluate the grading and distribution of lesions among patients undergoing biopsy. Radiologists' and urologists' performance and variability in prostate cancer detection (positive predictive value) were assessed by the detection of PCa (GG ≥ 1) and of csPCa (GG ≥ 2) by either template or targeted biopsy among men with PI‐RADS 3‐5 lesions. The Chi‐square test was used to evaluate for significant unadjusted differences between individuals.

Multivariable logistic regression (MVR) models evaluated the associations of clinical and MRI parameters with detection of PCa and of csPCa found on biopsy. The respective impact of radiologists and urologists to prostate cancer diagnosis was then evaluated by adding these variables to the base MVR models. Individual providers were evaluated relative to each other with odds ratios generated based on the (1) highest performing peer as reference for statistical significance and the (2) median performing peer as reference for bar graph visualisations. As an alternative measure of performance, observed probabilities were compared with predicted probabilities by individual provider. Impact of experience and case volume was evaluated by comparing the highest volume performers with the lowest based on median case volumes. Area under the curve (AUC) comparisons between MVR models assessed the relative impact of variability on discrimination for the outcome of PCa detection. All statistics were performed using STATA version 15.0 (STATA Corp, College Station, TX).^13^

## 3 RESULTS

### 3.1 Baseline characteristics

A total of 865 patients were included who underwent their first MRI with subsequent TRUS‐guided fusion prostate biopsy between 2015 and 2020 (Table 1). Of these men, 132 (15.3%) identified as African‐American, 194 (22.4%) reported a family history of prostate cancer and 469 (54.2%) were biopsy‐naïve. The median PSA density was 0.12 ng/mL/cc.

**TABLE 1:** Clinical and prostate MRI characteristics.

		Overall cohort	
		Median/*N*	IQR/(%)
*N*		865	‐
Age		66.0	60.8–70.2
Family history of prostate cancer	Yes	194	(22.4)
	No	667	(77.1)
	Unknown	4	(0.5)
Race	Caucasian	588	(68.0)
	Hispanic	53	(6.1)
	Asian	31	(3.6)
	African‐American	132	(15.3)
	Other/Unknown	61	(7.1)
DRE	Positive	84	(9.7)
	Negative	747	(86.4)
	Unknown	34	(3.9)
Prior negative biopsy	Yes	396	(45.8)
	No	469	(54.2)
PSA		6.4	4.8–9.4
PSAD		0.12	0.08–0.18
Prostate volume (cc)		52.0	37.0–76.4
Highest PI‐RADS lesion^a^	1 to 2	24	(2.8)
	3	309	(35.7)
	4	359	(41.5)
	5	173	(20.0)
Total number of PI‐RADS lesions	1	406	(46.9)
	2	296	(34.2)
	3	138	(16.0)
	≥4	25	(2.9)

Abbreviations: DRE, digital rectal exam; IQR, interquartile range; PSA, prostate‐specific antigen; PSAD, PSA density; PI‐RADS, Prostate Imaging‐Reporting and Data System.

^a^ Five patients were scored by PI‐RADS v2.1.

### 3.2 PI‐RADS grade and number of MRI lesions

**FIGURE 1:**
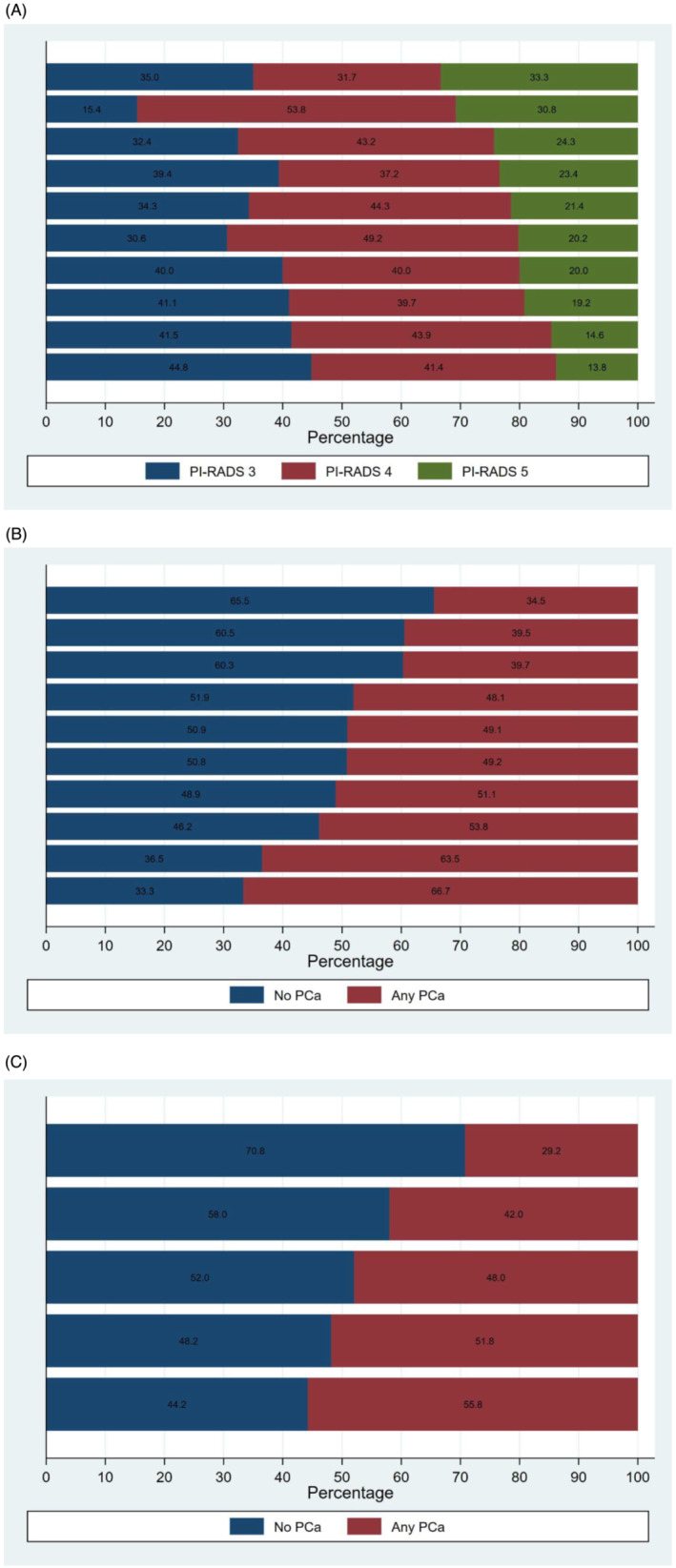
Variation in Prostate Imaging‐Reporting and Data System (PI‐RADS) grading by (A) radiologists and variation in prostate cancer detection by (B) radiologists and (C) urologists.

Twenty‐four (2.8%) patients receiving biopsy had a highest grade of PI‐RADS 1 or 2, 309 (35.7%) PI‐RADS 3, 359 (41.5%) PI‐RADS 4 and 173 (20.0%) PI‐RADS 5. Of the 841 studies with PI‐RADS ≥3 lesions, 98.1% were interpreted by 10 individual radiologists with a median case volume per radiologist of 65 (range 13 to 193) (Table 2). Although there was some variation, the relative frequency of PI‐RADS grades was similar based on Chi‐squared test for individual radiologists ranging from 15.4% to 44.8% of lesions graded PI‐RADS 3, 31.7% to 53.8% graded PI‐RADS 4 and 13.8% to 33.3% graded PI‐RADS 5 (*p* = 0.387) (Figure 1A). Conversely, detection of multifocality varied significantly by radiologist: Solitary lesions were identified between 24.1% and 68.3% of cases, two lesions between 23.3% and 40.7% of cases and three or more lesions between 8.3% and 48.3% (*p* < 0.001).

**TABLE 2:** Highest PI‐RADS and total number of PI‐RADS lesions by radiologist.

		Highest PI‐RADS lesion							Total number of PI‐RADS lesions							
		3	(%)	4	(%)	5	(%)	*p*‐value	1	(%)	2	(%)	3+	(%)	*p*‐value	Total
Radiologist^a^	#1	59	(30.6)	95	(49.2)	39	(20.2)	0.387	67	(34.7)	72	(37.3)	54	(28.0)	<0.001	193
	#2	62	(41.1)	60	(39.7)	29	(19.2)		71	(47.0)	55	(36.4)	25	(16.6)		151
	#3	51	(41.5)	54	(43.9)	18	(14.6)		55	(44.7)	50	(40.7)	18	(14.6)		123
	#4	37	(39.4)	35	(37.2)	22	(23.4)		43	(45.7)	37	(39.4)	14	(14.9)		94
	#5	24	(34.3)	31	(44.3)	15	(21.4)		43	(61.4)	18	(25.7)	9	(12.9)		70
	#6	21	(35.0)	19	(31.7)	20	(33.3)		41	(68.3)	14	(23.3)	5	(8.3)		60
	#7	22	(40.0)	22	(40.0)	11	(20.0)		26	(47.3)	22	(40.0)	7	(12.7)		55
	#8	12	(32.4)	16	(43.2)	9	(24.3)		19	(51.4)	11	(29.7)	7	(18.9)		37
	#9	13	(44.8)	12	(41.4)	4	(13.8)		7	(24.1)	8	(27.6)	14	(48.3)		29
	#10	2	(15.4)	7	(53.8)	4	(30.8)		4	(30.8)	4	(30.8)	5	(38.5)		13
	Other	6	(37.5)	8	(50.0)	2	(12.5)		11	(68.8)	2	(12.5)	3	(18.8)		16
	Total	309	‐	359	‐	173	‐		387	‐	293	‐	161	‐		841

Abbreviation: PI‐RADS, Prostate Imaging‐Reporting and Data System.

^a^ Radiologist numbering is based on case volume in this table and does not necessarily correspond to prostate cancer detection position in Table 3 to preserve anonymity.

Patients presenting to urologists for biopsy were similar in their PI‐RADS assessment as the distribution of PI‐RADS grades did not demonstrate significant variability (*p* = 0.577). The number of PI‐RADS lesions per patient was also similar across urologists (*p* = 0.459).

### 3.3 Prostate cancer detection

Five urologists performed 98.3% of the 865 biopsies, with case volumes ranging from 50 (5.8%) to 373 (43.1%) patients (median 113). PCa was detected in 417 (48.2%) patients, and csPCa was diagnosed in 285 (32.9%) patients. PCa and csPCa detection by PI‐RADS were 20.8% and 8.3% for PI‐RADS 1‐2, 23.3% and 9.4% for PI‐RADS 3, 54.6% and 37.3% for PI‐RADS 4 and 83.2% and 69.4% for PI‐RADS 5.

The rate of prostate cancer detection (positive predictive value) varied significantly by the radiologists responsible for the MRI interpretation. The overall rate of PCa detection by radiologist ranged from 34.5% to 66.7% (*p* = 0.003) (Figure 1B), while the rate of csPCa varied between 17.2% and 50.0% (*p* = 0.001) (supporting information Figure S1A). Stratified by PI‐RADS, PCa detection rates were between 0% and 21.6% for PI‐RADS 3, 25.0% and 71.0% for PI‐RADS 4, and 55.6% and 95.0% for PI‐RADS 5. Stratified by biopsy status, the rate of PCa detection for radiologists ranged from 40.0% to 80.0% for biopsy‐naïve patients and 24.8% to 44.1% for prior negative biopsy patients (supporting information Figure S2A,B). Of note, three radiologists had no cases of prostate cancer identified from PIRADS 3 lesions.

Prostate cancer detection by urologist varied from 29.2% to 55.8% (p = 0.01) (Figure 1C), but csPCa detection demonstrated less variability (24.6% to 39.8%, p = 0.36) (supporting information Figure S1B). PCa detection stratified by PI‐RADS was 9.1% to 15.7% for PI‐RADS 3, 31.8% to 49.1% for PI‐RADS 4, and 57.1% to 85.0% for PI‐RADS 5. Stratified by biopsy status, the rate of PCa detection for urologists ranged from 41.7% to 65.1% for biopsy‐naïve patients and 26.4% to 34.8% for prior negative biopsy patients (supporting information Figure S2C,D).

### 3.4 Multivariable analysis

Baseline MVR models were constructed for any PCa detection based on combined systematic and targeted biopsy. Positively associated factors included age, family history of prostate cancer, increased PSA density and PI‐RADS 4 and 5 lesions. AUC for the base model was 85.8%. Inclusion of radiologists in the MVR models improved AUC performance relative to the core clinical parameters for PCa detection from 85.8% to 86.6% (*p* = 0.017). Inclusion of urologists did not significantly improve the AUC for PCa (*p* = 0.22).

**FIGURE 2:**
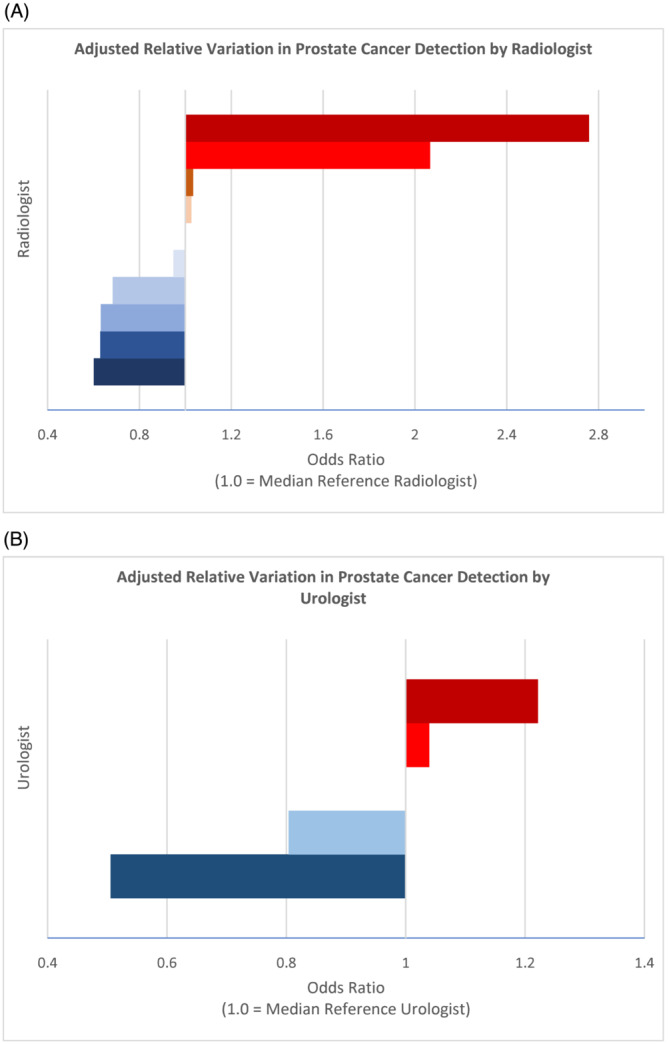
Adjusted relative variation in prostate cancer detection by (A) radiologist and (B) urologist. Median individual is set as the reference for visualisation.

Analysis of interrater variability in PCa detection demonstrated significant performance variability with a fourfold to fivefold adjusted difference between the highest‐performing radiologist relative to the lowest‐performing peer (OR 0.22, 95% CI 0.10–0.47, *p* < 0.001) (Table 3 and Figure 2). There was less variation in PCa detection by urologist although there was a statistically significant difference comparing the lowest performing peer to the highest (OR 0.41, 95% CI 0.19–0.91, *p* = 0.028). Binary classifications by practice volume did not demonstrate statistically significant differences when the top five radiologists were compared with the bottom 5 (0.94 (95% CI 0.61–1.45, *p* = 0.79) nor when the top two urologists were compared with the bottom 3 (OR 1.33 (95% CI 0.89–1.98, *p* = 0.16). As an alternative measure of performance, observed probabilities were compared with predicted probabilities from the baseline model for each provider. The degree and rank order of variation was similar to the model in Table 3, ranging from an absolute difference of +2.9% to −8.2% for PCa detection among urologists (relative +5.9% to −21.9%) and +12.6% to −6.9% for PCa detection among radiologists (relative +24.8% to −14.8%) (supporting information Table S1).

**TABLE 3:** Multivariable logistic regression model for prostate cancer detection.

Variable		Multivariable			
		OR	95%CI		*p*‐value
			Low	High	
Age		1.06	1.03	1.09	<0.001
Race	Caucasian	REF	‐	‐	‐
	Hispanic	0.82	0.37	1.79	0.612
	Asian	0.15	0.05	0.45	0.001
	African‐American	1.51	0.89	2.57	0.126
	Other/Unknown	0.95	0.45	2.01	0.894
Family history of PCa	No	REF	‐	‐	‐
	Yes	1.54	1.01	2.36	0.047
DRE	Negative	REF	‐	‐	‐
	Positive	0.70	0.37	1.34	0.286
	Unknown	1.84	0.59	5.79	0.297
Prior negative biopsy	No	REF	‐	‐	‐
	Yes	0.35	0.24	0.52	<0.001
PSAD^a^	(per 1 unit)	2.10	1.50	2.94	<0.001
Prostate volume (cc)	(per 1 cc)	0.98	0.97	0.98	<0.001
Highest PI‐RADS lesion^a^	1 to 2	1.14	0.35	3.71	0.825
	3	REF	‐	‐	‐
	4	3.74	2.50	5.62	<0.001
	5	14.07	4.07	48.65	<0.001
Urologist^b^	#1	REF	‐	‐	‐
	#2	0.85	0.55	1.31	0.463
	#3	0.82	0.45	1.48	0.509
	#4	0.66	0.29	1.49	0.316
	#5	0.41	0.19	0.91	0.028
Radiologist^b^	#1	REF	‐	‐	‐
	#2	0.75	0.28	2.03	0.570
	#3	0.37	0.17	0.84	0.018
	#4	0.37	0.14	0.98	0.046
	#5	0.36	0.15	0.86	0.021
	#6	0.34	0.16	0.76	0.008
	#7	0.25	0.07	0.82	0.023
	#8	0.23	0.08	0.67	0.007
	#9	0.23	0.05	1.11	0.068
	#10	0.22	0.10	0.47	<0.001

Abbreviations: 95%CI, 95% Confidence Interval; DRE, digital rectal exam; OR, odds ratio; PCa, prostate cancer; PI‐RADS, Prostate Imaging‐Reporting and Data System; PSA, prostate‐specific antigen; PSAD, PSA density.

^a^ Logarithmically transformed (natural log).

^b^ Relative to the highest performing provider; radiologist numbering is based on prostate cancer detection in this table and does not necessarily correspond to case volume in Table 2 to preserve anonymity.

Models for csPCa demonstrated similar findings with a statistically significant improvement in AUC with the inclusion of radiologists to the MVR (87.1% to 87.8%, *p =* 0.038) and no statistically significant difference with the inclusion of urologists (*p =* 0.086). A sensitivity analysis based on an outcome for PCa on targeted biopsy alone instead of combined biopsy found similar relationships with statistically significant model improvement for inclusion of radiologists (87.3% to 88.0%, *p =* 0.021) but not urologists (*p =* 0.54). An additional multivariable model to assess the impact of time period adjusted for other factors suggested an improvement in any PCa detection (OR 1.64, 95% CI 1.06–2.55, *p =* 0.03) and csPCa detection (OR 1.57, 95% CI 1.00–2.48, *p =* 0.05) comparing 2018–2020 to 2015–2017.

## 4 DISCUSSION

Variability in radiologist interpretation is a common critique of MRI use for prostate cancer diagnosis.^2^, ^3^ Although we found that the overall distribution of PI‐RADS grading between radiologists was similar, PCa and csPCa detection rates varied significantly between individual radiologists, with positive predictive values ranging from 34.5% to 66.7% for any PCa and 17.2% to 50.0% for csPCa. Furthermore, MVR revealed a small but significant increase in AUC when including radiologists as an independent factor impacting PCa detection while inclusion of the urologist performing biopsy had less impact. Additionally, practice case volume as a measure of experience did not appear to impact performance. These findings suggest that interreader variability remains a critical opportunity for improvement in prostate cancer diagnosis.

PI‐RADS 3 grading may represent a significant area of variability between radiologists that translates to differences in prostate cancer detection. A meta‐analysis of interreader agreement using PI‐RADS v2 demonstrated only moderate agreement for PI‐RADS ≥3 (pooled *κ* = 0.57), while substantial agreement for PI‐RADS ≥4 (pooled *κ* = 0.61).^8^ Previous MVR studies found that PI‐RADS 4 and 5 grades remained the greatest predictors of cancer diagnosis with biopsy, yet PI‐RADS 3 did not predict PCa detection.^3^, ^10^, ^12^ In this study, despite the lack of statistical disparity in the overall distribution of PI‐RADS grades, there was a relatively broad distribution of PI‐RADS 3 lesions across radiologists (15.4% to 44.8% of PI‐RADS ≥3 lesions) and differences in relative cancer detection rates (0% for three individuals yet 21.6% for another). These findings implicate PI‐RADS 3 as an area of disagreement or inconsistency, despite the efforts of PI‐RADS v2 to standardise radiologists' interpretation and emerging predictive tools.^14^

Inconsistency in PI‐RADS 3 grading is largely responsible for concerns over the use of MRI alone as a triage test in the PCa diagnostic pathway. While the PROMIS trial reported high sensitivity of MRI for csPCa, Sonn et al. reported 24% (22/90) of patients graded as PI‐RADS 1‐2 harboured csPCa on biopsy and the individual false negative rate varied between 13% and 60% across radiologists.^2^, ^10^ Unfortunately, many patients at our institution with negative MRI may not have undergone prostate biopsy limiting their representation in our sample focused primarily on fusion biopsy. Importantly, some patients excluded from biopsy may still harbour undetected csPCa and have a delayed diagnosis attributable to MRI interpretation. Early studies have suggested that dedicated reader education improves accuracy and confidence in prostate MRI interpretation, while computer aided diagnosis may improve sensitivity in detecting peripheral zone lesions.^15^, ^16^ At our centre, regular quality improvement sessions reviewing MRI and feedback after biopsy between radiologists and urologists were conducted during the early experience with prostate MRI which may have contributed to the improved PCa and csPCa detection we observed in recent years. Other considerations such as advanced serum and tissue biomarkers, prior biopsy status and validated risk calculators may all play a role in improving decisions regarding prostate biopsy to optimise detection of csPCa.^17^, ^18^

The impact of individual urologists on PCa detection appears to be significantly less pronounced than radiologists. While there was demonstrable variability in the detection of any PCa, only one urologist underperformed relative to the highest performer; additionally, there was no statistically significant difference in the potentially more important outcome of csPCa detection. Case volume experience between urologists also did not appear to significantly impact PCa detection. In an analysis by Stabile et al., they suggested that the learning curve to perform MRI‐fusion biopsies was between 60 and 80 cases.^19^ Interestingly, the use of MRI‐fusion in their study was associated with significantly higher rates of csPCa detection compared with cognitive‐fusion (57% vs. 36%, *p =* 0.002).^19^ While all urologists in our study were experienced in prostate biopsy, the use of MRI‐fusion was introduced during the study period such that the learning curve of the targeted biopsy technique is captured within the data. The lack of significant performance variability in csPCa detection suggests that real‐time image guidance may standardise performance and reduce the learning curve.

As this study was performed at a tertiary academic referral centre, generalisations may be limited. However, the presence of impactful interreader variability among radiology specialists underscores that greater variability may exist within low‐volume centres and may produce a proportional impact on PCa detection. Similarly, the study contained relatively few urologists with a broad range of practice volumes. Small provider groups likely reflect the reality for most practices, where wide distribution of patients may amplify differences in cancer detection between high and low performers. Regarding study design, only single radiologist interpretations were performed for MRI, limiting the ability to study interobserver agreement. This is also largely a study of patients with positive MRI findings and does not sufficiently study potential cancer detection in patients with negative MRI.

Despite its limitations, the study provides a robust analysis of radiologists' interpretation of MRI for PI‐RADS and lesions as well as downstream impact on PCa detection. It also identifies variability in urologists' biopsy execution but that the impact on PCa detection was more driven by radiologists' interpretation. The measured effect on PCa detection underscores the need to implement quality improvement efforts through continued evaluation of PI‐RADS grading, educational interventions and feedback through relationships between urologists and radiologists to further improve care for patients with a clinical suspicion for PCa.

## 5 CONCLUSIONS

Variability among radiologists in PI‐RADS grading significantly impacts the detection of PCa and csPCa based on MRI‐TRUS fusion prostate biopsy making it a key area for quality improvement efforts. While there is variability across urologists in performance of prostate biopsy, the impact on PCa detection is minimal compared with MRI interpretation. Notably, cancer detection was improved in more recent years.

## AUTHOR CONTRIBUTIONS

**Hiten D. Patel**: Conceptualization; methodology; software; formal analysis; data curation; writing—original draft; writing—reviewing and editing. **Whitney R. Halgrimson**: Conceptualization; writing—original draft; writing—reviewing and editing. **Sarah E. Sweigert**: Conceptualization; writing—reviewing and editing. **Steven M. Shea**: Conceptualization; data curation; writing—reviewing and editing; supervision; funding acquisition. **Thomas M. T. Turk**: Writing—reviewing and editing; supervision. **Marcus L. Quek**: Writing—reviewing and editing; supervision. **Alex Gorbonos**: Writing—reviewing and editing; supervision. **Robert C. Flanigan**: Writing—reviewing and editing; supervision. **Ari Goldberg**: Writing—reviewing and editing; supervision. **Gopal N. Gupta**: Conceptualization; writing—reviewing and editing; supervision.

## CONFLICT OF INTEREST STATEMENT

None.

## Supporting information


**Table S1.** Comparison of average observed probability to predicted probability for detection of prostate cancer by individual urologists and radiologists.

**Figure S1.** Variation in Clinical Significant Prostate Cancer Detection by (A) Radiologists and (B) Urologists.

**Figure S2.** Variation in Prostate Cancer Detection by (A) Radiologists for Biopsy Naïve Patients, (B) Radiologists for Prior Negative Biopsy Patients, (C) Urologists for Biopsy Naïve Patients, and (D) Urologists for Prior Negative Biopsy Patients. One radiologist was excluded due to having <14 cases in the biopsy naive and <14 cases in the prior negative groups.
