# Supplementary material for: Variability in prostate cancer detection among radiologists and urologists using MRI fusion biopsy
Source: BJUI Compass. 2023 Oct 8;5(2):304–12. doi: 10.1002/bco2.294 (PMC10869647; doi:10.1002/bco2.294)
Supplement: Supplementary file 1 — Table S1. Comparison of average observed probability to predicted probability for detection of prostate cancer by individual urologists and radiologists. [file BCO2-5-304-s001.docx]

Supplemental Table 1. Comparison of average observed probability to predicted probability for detection of prostate cancer by individual urologists and radiologists.

|  | **Number** | **Predicted Probability** | **Observed Probability** | **Absolute Difference** |
| --- | --- | --- | --- | --- |
| **Urologist** | #1 | 0.489 | 0.518 | 0.029 |
|  | #2 | 0.564 | 0.558 | -0.006 |
|  | #3 | 0.491 | 0.480 | -0.011 |
|  | #4 | 0.444 | 0.420 | -0.024 |
|  | #5 | 0.374 | 0.292 | -0.082 |
| **Radiologist** | #1 | 0.509 | 0.635 | 0.126 |
|  | #2 | 0.580 | 0.667 | 0.087 |
|  | #3 | 0.494 | 0.511 | 0.017 |
|  | #4 | 0.479 | 0.492 | 0.013 |
|  | #5 | 0.478 | 0.481 | 0.003 |
|  | #6 | 0.496 | 0.491 | -0.005 |
|  | #7 | 0.390 | 0.345 | -0.045 |
|  | #8 | 0.596 | 0.538 | -0.058 |
|  | #9 | 0.454 | 0.395 | -0.059 |
|  | #10 | 0.466 | 0.397 | -0.069 |
