# Supplementary material for: Variability in prostate cancer detection among radiologists and urologists using MRI fusion biopsy
Source: BJUI Compass. 2023 Oct 8;5(2):304–12. doi: 10.1002/bco2.294 (PMC10869647; doi:10.1002/bco2.294)

Supplemental Figure 1. Variation in Clinical Significant Prostate Cancer Detection by (A) Radiologists and (B) Urologists.

A)


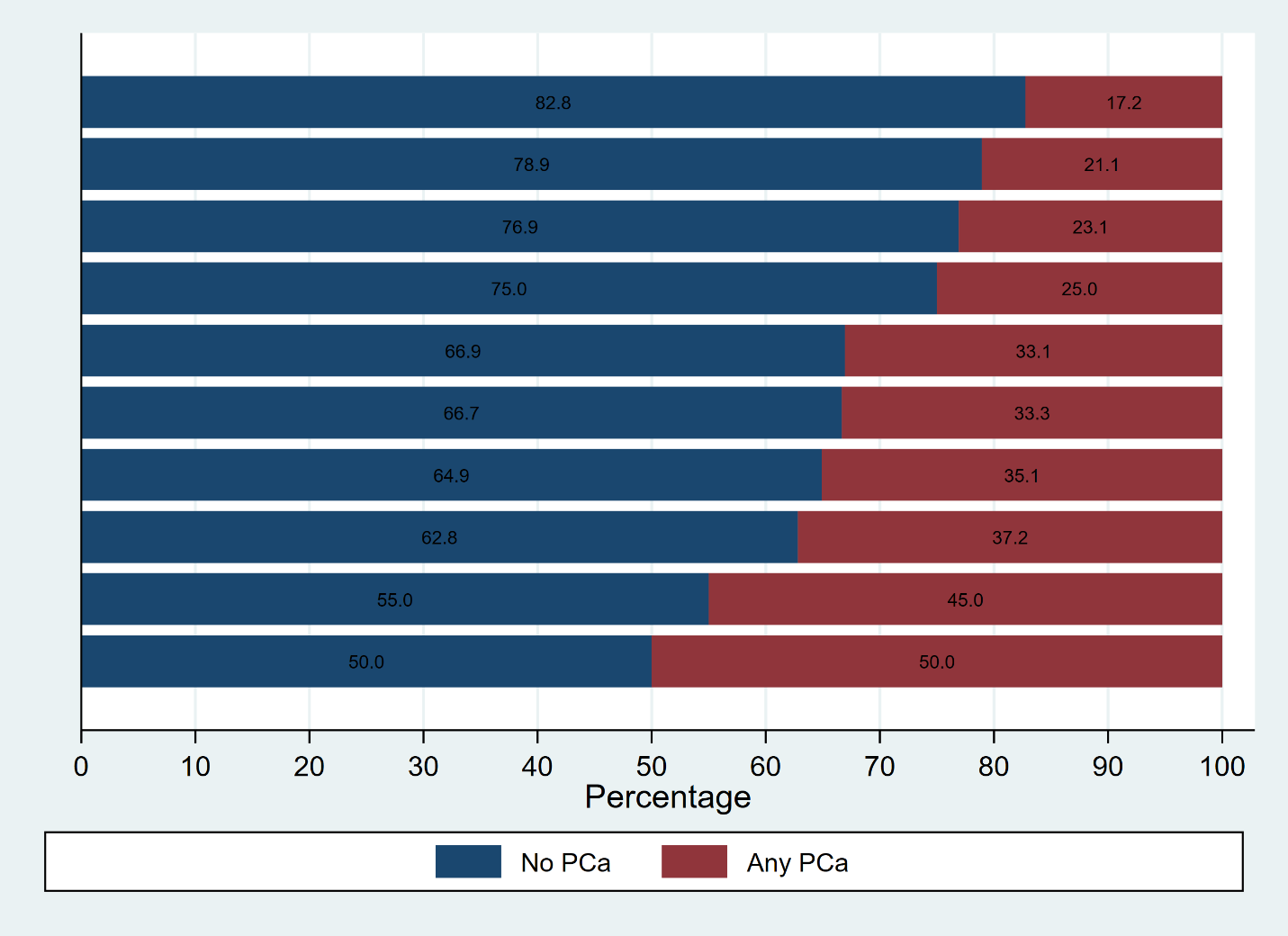


B)


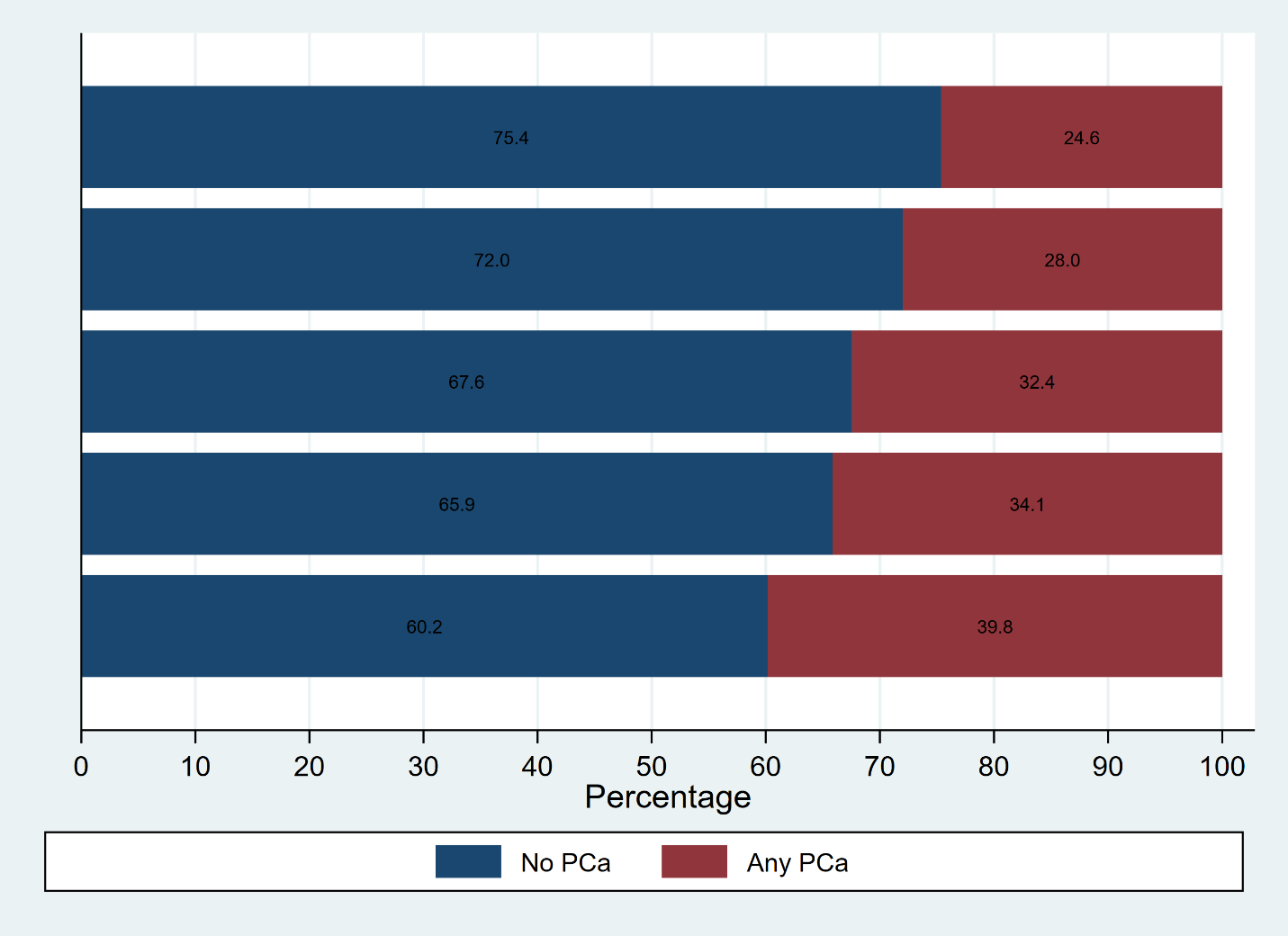

Supplement: Supplementary file 2 — Figure S1. Variation in Clinical Significant Prostate Cancer Detection by (A) Radiologists and (B) Urologists. [file BCO2-5-304-s002.docx]
