# Supplementary material for: Variability in prostate cancer detection among radiologists and urologists using MRI fusion biopsy
Source: BJUI Compass. 2023 Oct 8;5(2):304–12. doi: 10.1002/bco2.294 (PMC10869647; doi:10.1002/bco2.294)

Supplemental Figure 2. Variation in Prostate Cancer Detection by (A) Radiologists for Biopsy Naïve Patients, (B) Radiologists for Prior Negative Biopsy Patients, (C) Urologists for Biopsy Naïve Patients, and (D) Urologists for Prior Negative Biopsy Patients. One radiologist was excluded due to having <14 cases in the biopsy naive and <14 cases in the prior negative groups.

A)


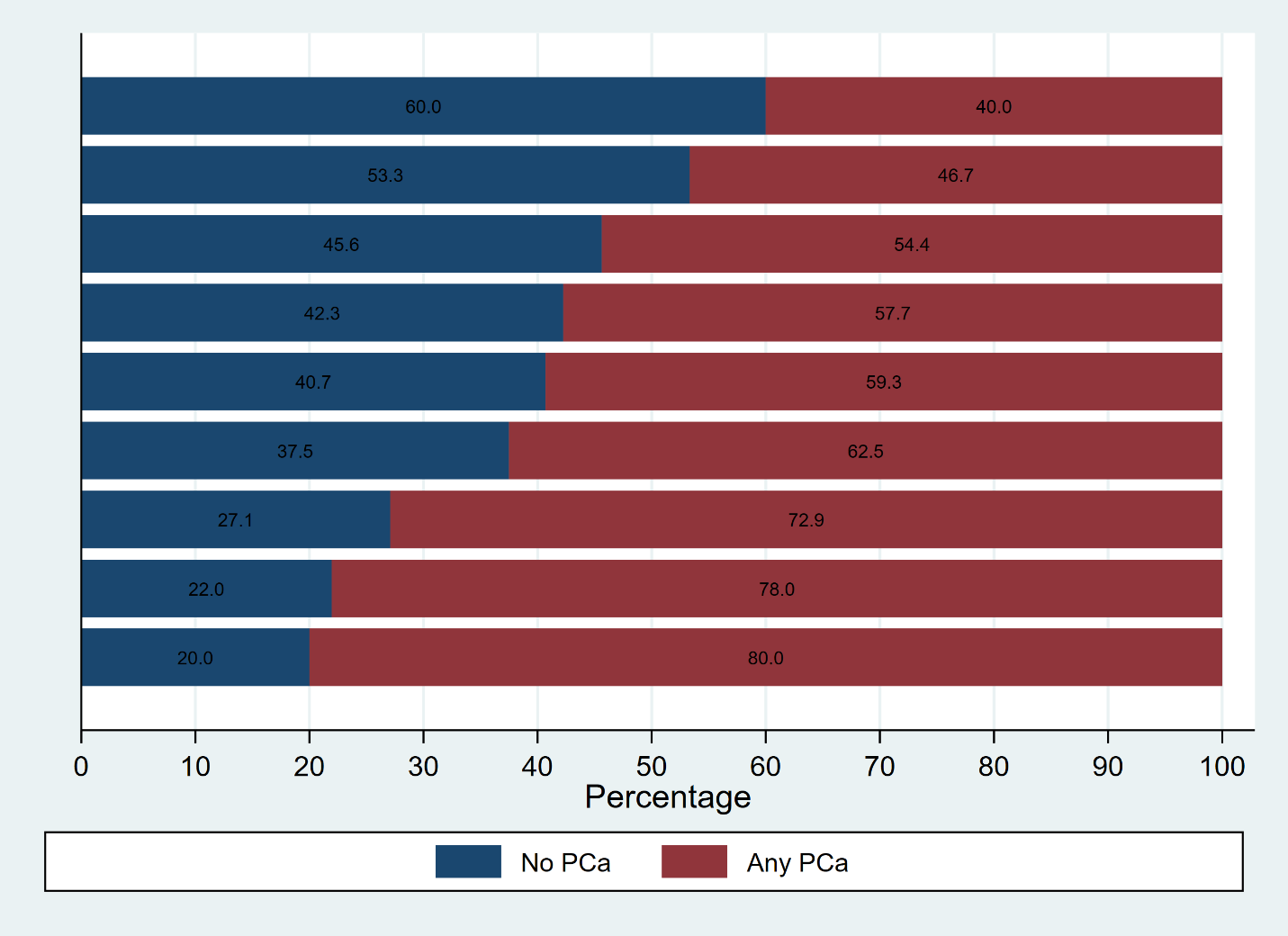


B)


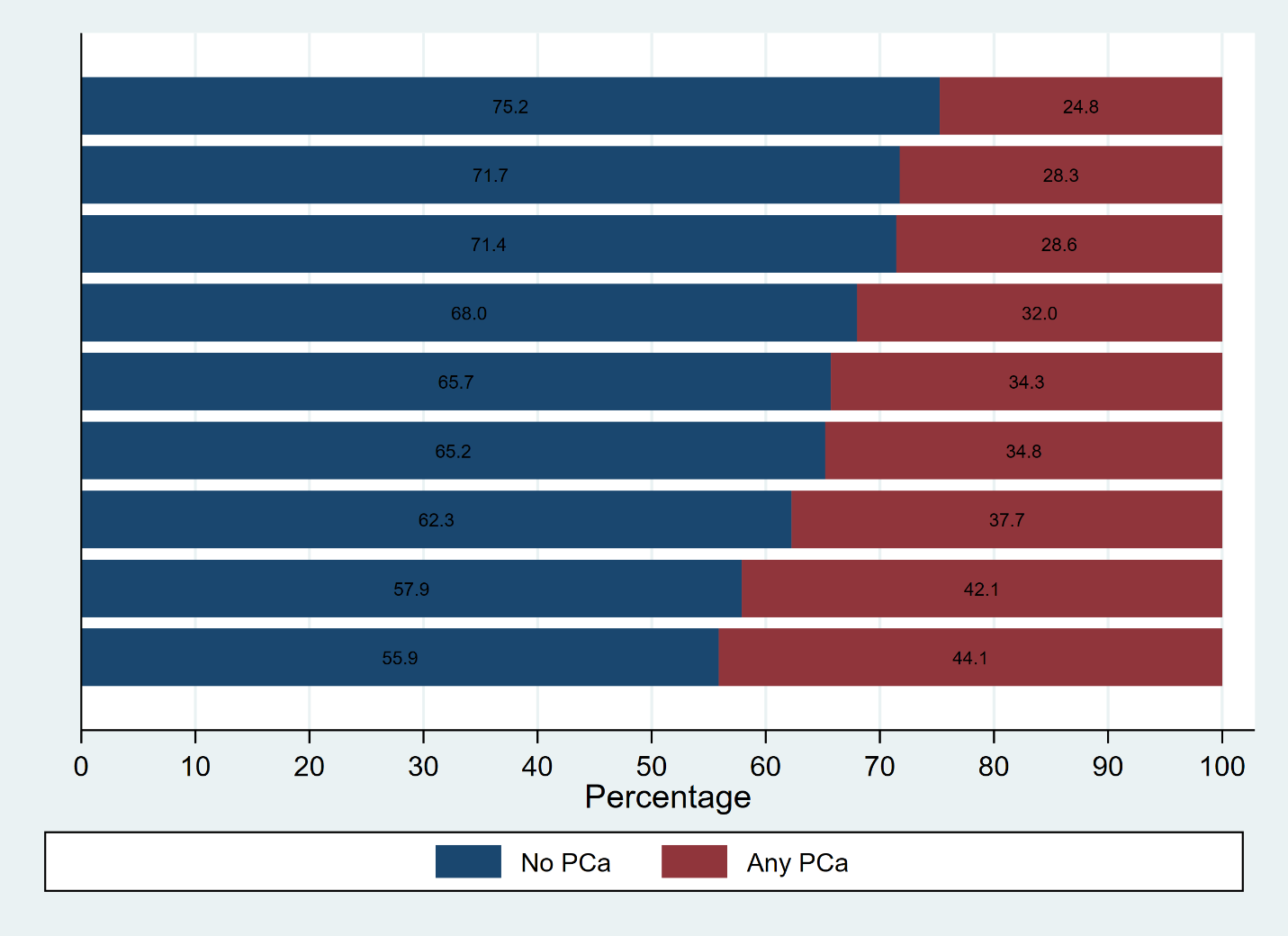


C)


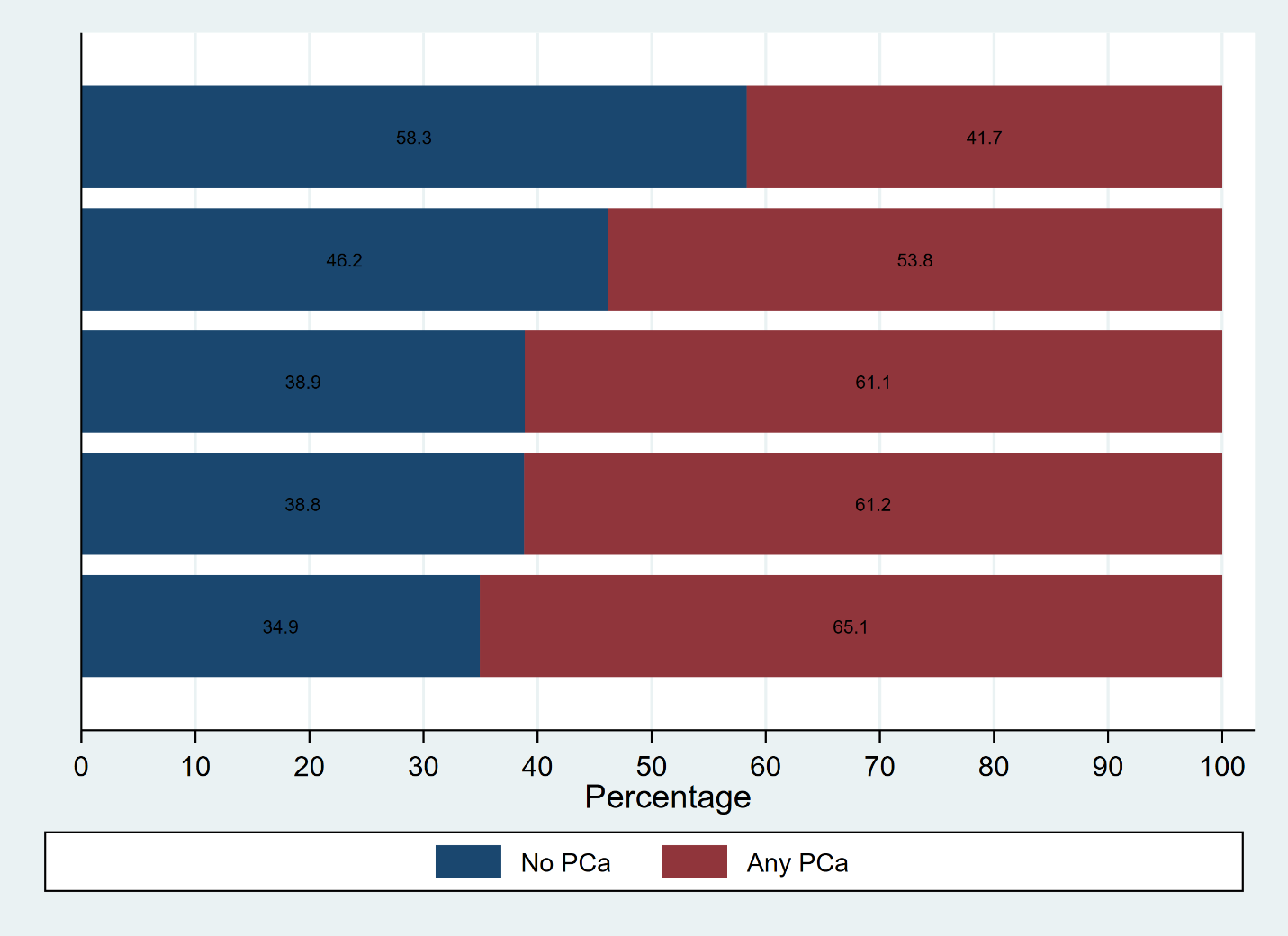


D)


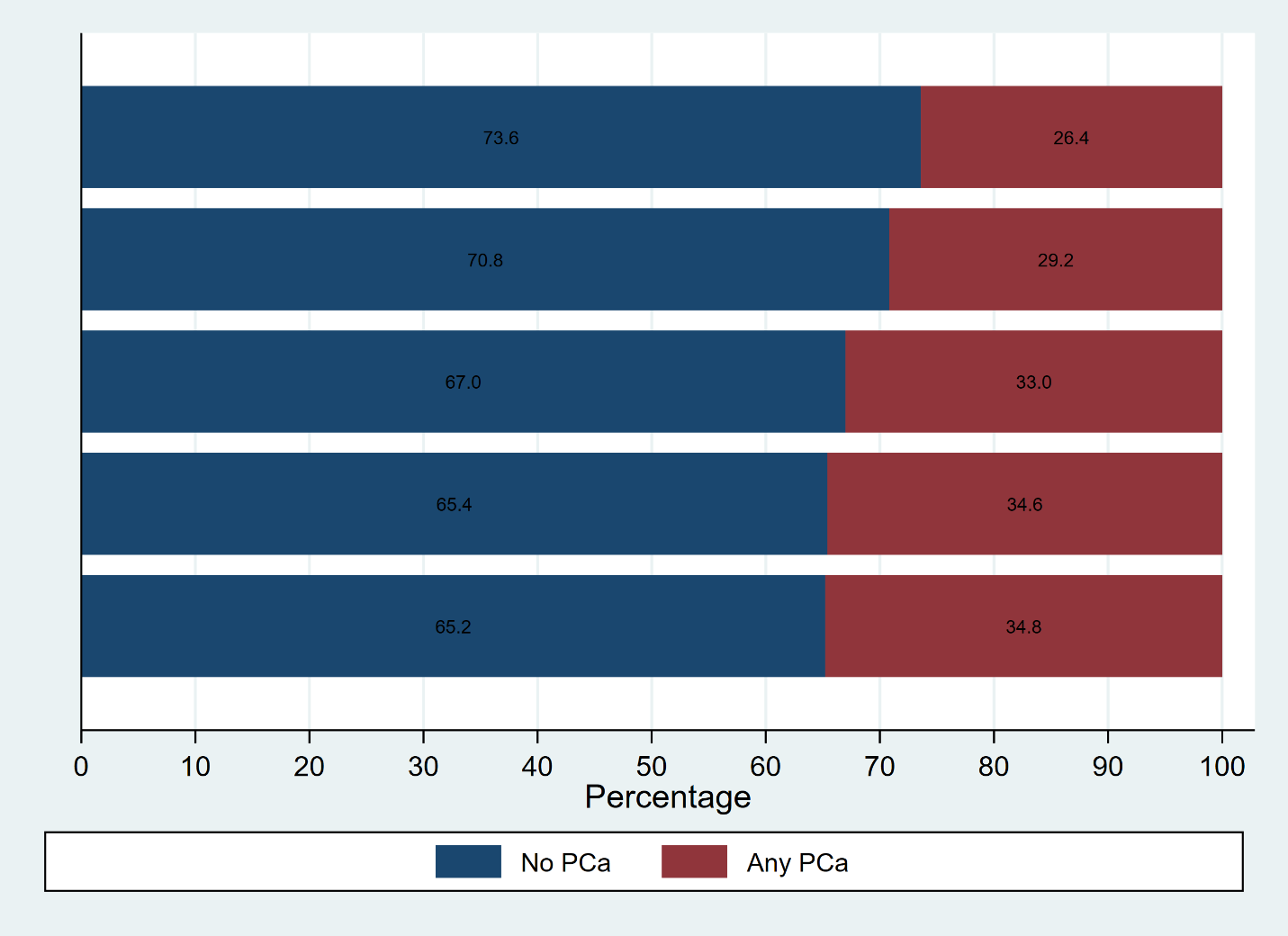

Supplement: Supplementary file 3 — Figure S2. Variation in Prostate Cancer Detection by (A) Radiologists for Biopsy Naïve Patients, (B) Radiologists for Prior Negative Biopsy Patients, (C) Urologists for Biopsy Naïve Patients, and (D) Urologists for Prior Negative Biopsy Patients. One radiologist was excluded due to having <14 cases in the biopsy naive and <14 cases in the prior negative groups. [file BCO2-5-304-s003.docx]
